# See, Saw, Marjorie Daw (Poetry)

**Published:** 1876-07

**Authors:** Clara Doty Bates


BY MRS. CLARA DOTY BATES.

I THINK of a pictured saint,
With a halo round the hair,
As she sits so motherly, grave, and quaint,
In her little rocking-chair.
Her dolly is on her breast,
And her tender-lidded eyes
Gaze softly downward on its rest—
Loving, Madonna-wise.
What is the lullaby she sings,
As back and forth she swings, and swings?
"See saw,
Marjorie Daw!"

Not a dimpled baby at all,
Is this which her arms caress,
But it bears the mark of many a fall,
And is sadly scant of dress.
And the washed-out cheeks display
Proof that it must have lain,
After some tired summer play,
Out over night in the rain.
Yet doth the little mother sing
Tenderly to the battered thing,
"See, saw,
Marjorie Daw!"

What words for a cradle song!
But I know the sleepy sign:
She will croon awhile, and then ere long
Will leave her chair for mine;
And her voice will sink away
To a feeble nestling's caw,
Till the little tongue can scarcely say—
"See, saw, Majorie Daw!"
Now, back and forth we swing, and swing,
But it is only I who sing—
"See, saw,
Marjorie Daw!"

—*St. Nicholas for June.*

